# Genomic Features of Newly Diagnosed Large B-cell Lymphoma with or without Subsequent Disease Progression

**DOI:** 10.1158/2767-9764.CRC-24-0337

**Published:** 2024-11-13

**Authors:** Daniel J. Landsburg, Jennifer J.D. Morrissette, Sunita D. Nasta, Stefan K. Barta, Stephen J. Schuster, Elise A. Chong, Jakub Svoboda, Ashley Barlev, Adam Bagg, Salvatore F. Priore

**Affiliations:** 1 Lymphoma Program, Abramson Cancer Center, University of Pennsylvania, Philadelphia, Pennsylvania.; 2 Department of Pathology and Laboratory Medicine, University of Pennsylvania, Philadelphia, Pennsylvania.

## Abstract

Genomic analysis has the potential to both risk-stratify and inform management of patients diagnosed with large B-cell lymphomas (LBCL). We analyzed cases of patients with newly diagnosed LBCL treated with standard immunochemotherapy from three publicly available cohorts of patients on which FISH and next-generation sequencing were performed to determine the frequency of genomic alterations based upon the development of disease progression. Cases from 698 patients were analyzed, with 201 experiencing disease progression and 497 experiencing no disease progression by 24 months after diagnosis. When analyzing for the presence of *MYC* rearrangement and *MYC*–*BCL2* dual-rearrangement/double-hit status, as well as variants predicted to result in alterations in protein function in 15 genes common to next-generation sequencing panels from all three cohorts, only *MYC* rearrangement and *TP53* mutation were associated with significantly higher odds of disease progression on multivariate analysis. Additionally, cases from patients who experienced disease progression demonstrated a high frequency of specific genomic alterations when analyzed by cohort or cell-of-origin classification by IHC when available. Individual genomic features of LBCL cases may predict for the development of disease progression in newly diagnosed patients treated with standard therapies, as well as occur at higher frequencies in cases of disease progression based upon geographic region and/or cell-of-origin status. These novel findings support efforts to evaluate genomic features as biomarkers for response to specific therapies in subsets of patients with LBCL who experience disease progression, which may lead to discovery of more effective treatment options.

**Significance:**

Genomic features of LBCL that can be detected by clinical laboratory assays may predict for resistance to first-line immunochemotherapy, as well as support the exploration of genomic features as biomarkers of response to therapies which could be offered to patients who experience disease progression.

## Introduction

Approximately one third of patients with newly diagnosed large B-cell lymphoma (LBCL) will not be cured following treatment with first-line immunochemotherapy. Numerous treatment options are currently available and under investigation for patients with relapsed/refractory (R/R) LBCL; however, it remains largely unclear about which patients will benefit from specific therapies.

Comprehensive genomic analysis, largely based on mutation analysis, can assign a genetic classification to the majority of those tumors from patients with newly diagnosed LBCL and risk-stratify survival outcomes following treatment with first-line immunochemotherapy (1, 2). However, it is not known if these genetic classifications are predictive of survival of patients with R/R LBCL following treatment with subsequent lines of therapy. Furthermore, there are limitations of comprehensive genomic analysis, including its inability to assign all cases to a specific genetic classification and the lack of availability in routine clinical practice. Additionally, LBCL genetic classification algorithms frequently include genetic variants without regard to their effect on protein function.

Next generation sequencing (NGS), which is available for clinical use in academic and commercial laboratories, can identify individual recurring genetic variants, and resources are available to determine the likelihood of pathogenicity of a given variant identified by NGS. As most R/R LBCL therapies are targeted toward a specific receptor or signaling pathway, it may be informative to identify individual genomic variants within LBCL tumors, which predict for alterations in protein function as these may predict for sensitivity or resistance to a specific agent.

We aimed to determine the frequency of genomic features based upon disease progression status from a large international series of patients with newly diagnosed LBCL in hopes of informing current management of this population and design of future clinical trials for those experiencing disease progression.

## Materials and Methods

### Case selection and categorization

**Figure 1:**
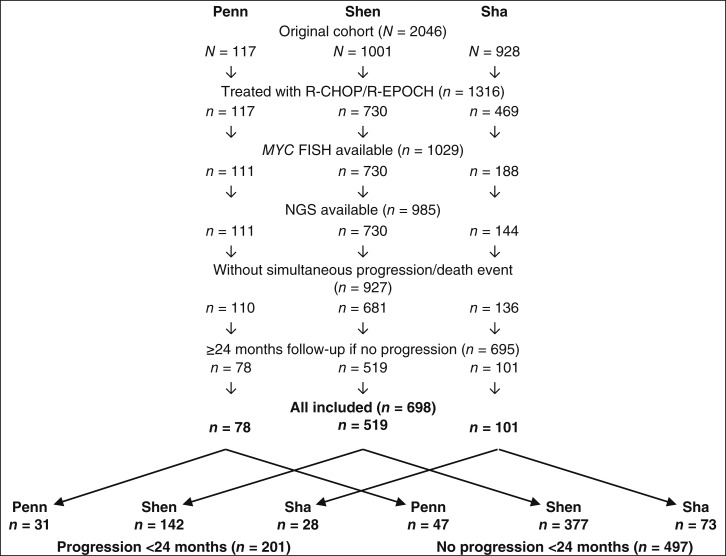
Case inclusion/exclusion.

Included cases were those from three series of patients with newly diagnosed LBCL whose tumor features as well as time to first relapse were made available through publication of one randomized clinical trial (Sha; ref. 3) and two retrospective series (Penn, Shen; refs. 4, 5), having been identified through search of PubMed as well as cBioPortal (www.cbioportal.org). The process for case selection is depicted in Fig. 1. Testing for cell of origin (COO), FISH and mutation analysis were performed as described in the original studies. Included FISH results were those for *MYC* rearrangement and *BCL2* rearrangement if *MYC* rearrangement was detected. Included genes were those that were sequenced in all three cohorts: *B2M*, *CD79B*, *CIITA*, *CREBBP*, *zeste homolog 2* (*EZH2*), *GNA13*, *MYD88*, *NOTCH1*, *NOTCH2*, *SOCS1*, *STAT3*, *TET2*, *TNFAIP3*, *TNFRSF14*, and *TP53*, and variants of these genes were analyzed for predicted change in protein function as per OncoKB (www.oncokg.org), as well as *TP53* loss-of-function mutations as previously described (4), with those variants predicted to result in change in protein function included in the analysis. Cases categorized as progression were those from patients reported to experience a progression event <24 months after initial diagnosis, whereas those categorized as no progression were those from patients not reported to experience a progression event <24 months after initial diagnosis with at least 24 months of follow-up in remission.

### Statistical analysis

ORs for disease progression were determined by logistic regression. Categorical variables were compared using the *χ*^2^ test. Statistical significance was defined as a two-tailed *P* value < 0.05. All statistical analyses were performed using Stata version 13 (StataCorp). This protocol was approved by the Institutional Review Board of the University of Pennsylvania.

### Data availability

The data generated in this study are available within the article and its supplementary data files.

## Results

Cases from 698 patients were included, with 201 (29%) patients experiencing progression and 497 (71%) patients experiencing no progression, as depicted in Fig. 1. There was no statistically significant difference among the proportions of progression cases by series (Penn 31/78, 40%; Shen 142/519, 30%; Sha 28/101, 28%; *P* = 0.08). The median length of follow-up for the entire cohort was 41.6 months and exceeded 24 months in all cohorts (Penn, 39.6 months; Shen, 44.5 months; Sha, 34.5 months).

**Figure 2:**
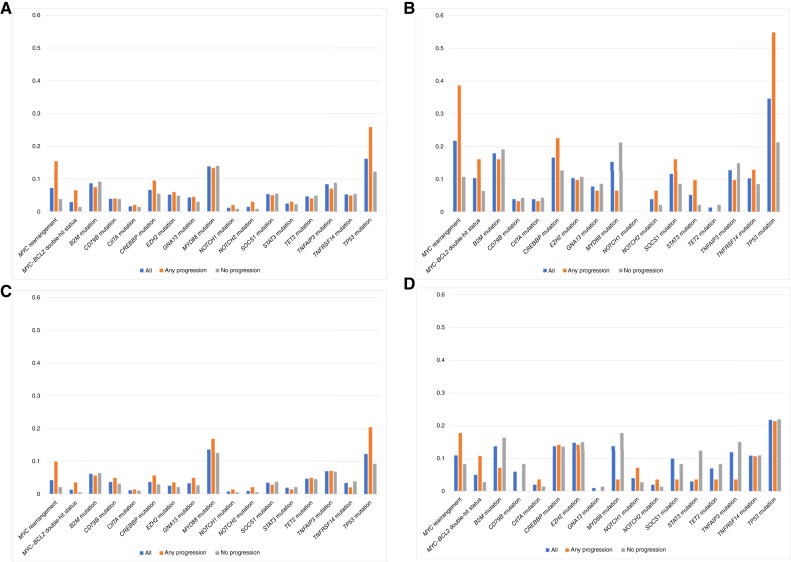
Frequency of genomic alterations for (**A**) all patients, (**B**) Penn cohort patients, (**C**) Shen cohort patients, and (**D**) Sha cohort patients.

For the entire cohort, the frequency of genomic alterations in all cases versus relapsed cases versus no-relapse cases is depicted in Fig. 2A, with the same distribution for the Penn, Shen, and Sha series depicted in Fig. 2B–D, respectively. Data for these figures, including International Prognostic Index (IPI) score, are listed in Table 1. For all cases in the entire cohort, *MYC* rearrangement was detected in 7% of cases and *MYC*–*BCL2* double-hit lymphoma (DHL) in 3% of cases, and the most frequently occurring mutations were in *TP53* (16%), *MYD88* (14%), and *B2M* (9%). For progression cases in the entire cohort, *MYC* rearrangement was detected in 15% of cases and *MYC*–*BCL2* DHL in 6% of cases, and the most frequently occurring mutations were in *TP53* (26%), *MYD88* (13%), *B2M* (9%), and *CREBBP* (9%). For no-progression cases in the entire cohort, *MYC* rearrangement was detected in 4% of cases and *MYC*–*BCL2* DHL in 1% of cases, and the most frequently occurring mutations were in *MYD88* (14%), *TP53* (12%), *B2M* (9%), and *TNFAIP3* (9%).

**Table 1:** Count of genomic alterations in all patients and by cohort

Factor	All patients		
	All *n* = 698 (%)	No progression *n* = 497 (%)	Progression *n* = 201 (%)
IPI ≥3	190 (27)	97 (20)	93 (46)
*MYC* rearrangement	50 (7)	19 (4)	31 (15)
*MYC*–*BCL2* double-hit status	20 (3)	7 (1)	13 (6)
*B2M* mutation	60 (9)	45 (9)	15 (7)
*CD79B* mutation	27 (4)	19 (4)	8 (4)
*CIITA* mutation	11 (2)	7 (1)	4 (2)
*CREBBP* mutation	46 (7)	27 (5)	19 (9)
*EZH2* mutation	36 (5)	24 (5)	12 (6)
*GNA13* mutation	30 (4)	15 (3)	9 (4)
*MYD88* mutation	97 (14)	70 (14)	27 (13)
*NOTCH1* mutation	8 (1)	4 (1)	4 (2)
*NOTCH2* mutation	10 (1)	4 (1)	6 (3)
*SOCS1* mutation	37 (5)	27 (5)	10 (5)
*STAT3* mutation	17 (2)	11 (2)	6 (3)
*TET2* mutation	32 (5)	24 (5)	8 (4)
*TNFAIP3* mutation	58 (8)	44 (9)	14 (7)
*TNFRSF14* mutation	37 (5)	27 (5)	10 (5)
*TP53* mutation	113 (16)	61 (13)	52 (26)

Factor	Penn cohort patients		
	All *n* = 78 (%)	No progression *n* = 47 (%)	Progression *n* = 31 (%)
IPI ≥3	35 (45)	19 (40)	16 (52)
*MYC* rearrangement	17 (22)	5 (11)	12 (39)
*MYC*–*BCL2* double-hit status	8 (10)	3 (6)	5 (16)
*B2M* mutation	14 (18)	9 (19)	5 (16)
*CD79B* mutation	3 (4)	2 (4)	1 (3)
*CIITA* mutation	3 (4)	2 (4)	1 (3)
*CREBBP* mutation	13 (17)	6 (13)	7 (23)
*EZH2* mutation	8 (10)	5 (11)	3 (10)
*GNA13* mutation	6 (8)	4 (9)	2 (6)
*MYD88* mutation	12 (15)	10 (21)	2 (6)
*NOTCH1* mutation	0	0	0
*NOTCH2* mutation	3 (4)	1 (2)	2 (6)
*SOCS1* mutation	9 (12)	4 (9)	5 (16)
*STAT3* mutation	4 (5)	1 (2)	3 (10)
*TET2* mutation	1 (1)	1 (2)	0
*TNFAIP3* mutation	10 (13)	7 (15)	3 (10)
*TNFRSF14* mutation	8 (10)	4 (9)	4 (13)
*TP53* mutation	27 (35)	10 (21)	17 (54)

Factor	Shen cohort patients		
	All *n* = 519 (%)	No progression *n* = 377 (%)	Progression *n* = 142 (%)
IPI ≥3	113 (22)	50 (13)	63 (44)
*MYC* rearrangement	22 (4)	8 (2)	14 (10)
*MYC–BCL2* double-hit status	7 (1)	2 (1)	5 (4)
*B2M* mutation	32 (6)	24 (6)	8 (7)
*CD79B* mutation	19 (4)	12 (3)	7 (5)
*CIITA* mutation	6 (1)	4 (1)	2 (1)
*CREBBP* mutation	19 (4)	11 (3)	8 (7)
*EZH2* mutation	13 (3)	8 (2)	5 (4)
*GNA13* mutation	17 (3)	10 (3)	7 (5)
*MYD88* mutation	71 (14)	47 (12)	24 (17)
*NOTCH1* mutation	4 (1)	2 (1)	2 (1)
*NOTCH2* mutation	5 (1)	2 (1)	3 (2)
*SOCS1* mutation	18 (3)	14 (4)	4 (3)
*STAT3* mutation	10 (2)	8 (2)	2 (1)
*TET2* mutation	24 (5)	17 (5)	7 (5)
*TNFAIP3* mutation	36 (7)	26 (7)	10 (7)
*TNFRSF14* mutation	18 (3)	15 (4)	3 (2)
*TP53* mutation	64 (12)	35 (9)	29 (20)

Factor	Sha cohort patients		
	All *n* = 101 (%)	No progression *n* = 73 (%)	Progression *n* = 28 (%)
IPI ≥3	42 (42)	28 (36)	14 (50)
*MYC* rearrangement	11 (11)	6 (8)	5 (18)
*MYC*–*BCL2* double-hit status	5 (5)	2 (3)	3 (11)
*B2M* mutation	14 (14)	12 (15)	2 (7)
*CD79B* mutation	6 (6)	6 (8)	0
*CIITA* mutation	2 (2)	1 (1)	1 (4)
*CREBBP* mutation	14 (14)	10 (13)	4 (14)
*EZH2* mutation	15 (15)	11 (14)	4 (14)
*GNA13* mutation	1 (1)	1 (1)	0
*MYD88* mutation	14 (14)	13 (17)	1 (4)
*NOTCH1* mutation	4 (4)	2 (3)	2 (7)
*NOTCH2* mutation	2 (2)	1 (1)	1 (4)
*SOCS1* mutation	10 (10)	6 (8)	1 (4)
*STAT3* mutation	3 (3)	9 (12)	1 (4)
*TET2* mutation	7 (7)	6 (8)	1 (4)
*TNFAIP3* mutation	12 (12)	11 (14)	1 (4)
*TNFRSF14* mutation	11 (11)	8 (10)	3 (11)
*TP53* mutation	22 (22)	16 (21)	6 (21)

**Figure 3:**
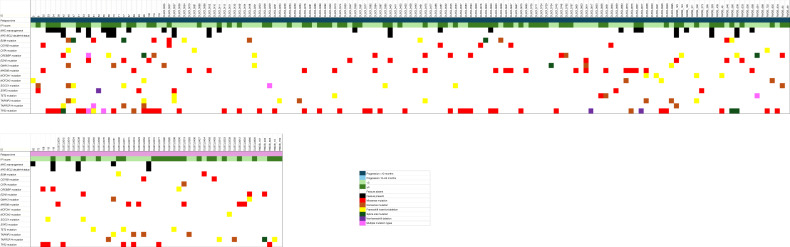
Genomic features of individual relapse cases.

A heat map including features listed in Table 1 for cases of progression is depicted in Fig. 3. A detailed description of genomic variants and other clinicopathologic information by case is listed in Supplementary Table S1.

The features listed in Table 1 were incorporated into logistic regression modeling to determine their association with odds of progression events. As listed in Supplementary Table S2, for the entire cohort, IPI score ≥3, *MYC* rearrangement, *MYC*–*BCL2* DHL, and *TP53* mutation were associated with significantly higher odds of progression on univariate analysis but only IPI score ≥3, *MYC* rearrangement, and *TP53* mutation on multivariate analysis. For the Penn series, *MYC* rearrangement and *TP53* mutation were associated with significantly higher odds of progression on both univariate and multivariate analyses. For the Shen series, IPI score ≥3, *MYC* rearrangement, *MYC*–*BCL2* DHL, and *TP53* mutation were associated with significantly higher odds of progression on univariate analysis but only IPI score ≥3 and *TP53* mutation on multivariate analysis. For the Sha series, no feature was associated with higher odds of progression on univariate analysis.

For the entire cohort, nearly all progression cases with *MYC* rearrangement (26/31, 84%) and *TP53* mutation (45/52, 87%) demonstrated disease progression by 12 months from diagnosis. However, more than half (91/151, 60%) of cases with disease progression by 12 months harbored neither *MYC* rearrangement nor *TP53* mutation.

**Figure 4:**
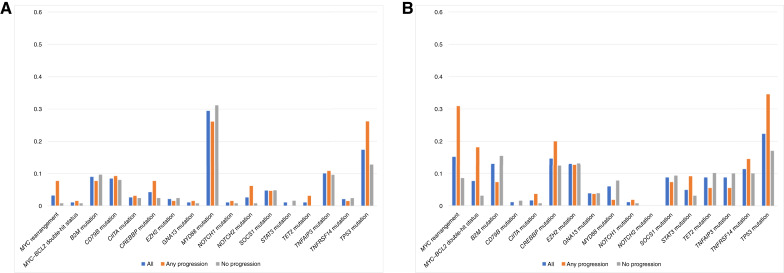
Frequency of genomic alterations for (**A**) non-GCB patients and (**B**) GCB patients.

Considering the fact that genomic alterations may vary by COO, we performed a subset analysis of cases (*n* = 374) for which COO was reported. Details of COO distribution of cases for the entire cohort and by series are listed in Supplementary Table S3. There was no statistically significant difference among the proportions of progression cases by COO (non–germinal center B (GCB) 65/190, 34% vs. GCB 55/184, 30%; *P* = 0.37). For the entire cohort with COO reported, the frequency of genomic alterations in all cases versus relapsed cases versus no-relapse cases by GCB and non-GCB COO is depicted in Fig. 4A and B, respectively. Data for these figures, including IPI score, are listed in Supplementary Table S4.

Additionally, the features listed in Table 1 were incorporated into logistic regression modeling to determine their association with odds of progression events for patients with GCB and non-GCB cases. As listed in Supplementary Table S5, for GCB cases, IPI score ≥3, *MYC* rearrangement, and *TP53* mutation were associated with significantly higher odds of progression on both univariate and multivariate analyses; for non-GCB cases, IPI score ≥3, *MYC* rearrangement, *MYC*–*BCL2* DHL, and *TP53* mutation were associated with significantly higher odds of progression on univariate analysis, but no factor remained significant on multivariate analysis. Finally, the percentage of genomic alterations in all progression cases, as well as progression cases subdivided by series and COO, is listed in Table 2.

**Table 2:** Percentage of genomic alterations in progression cases

Any progression	*MYC* rearrangement	*MYC*–*BCL2* double-hit status	*B2M* mutation	*CD79B* mutation	*CIITA* mutation	*CREBBP* mutation	*EZH2* mutation	*GNA13* mutation	*MYD88* mutation	*NOTCH1* mutation	*NOTCH2* mutation	*SOCS1* mutation	*STAT3* mutation	*TET2* mutation	*TNFAIP3* mutation	*TNFRSF14* mutation	*TP53* mutation
All (*n* = 201)	15.4%	6.5%	7.5%	4.0%	2.0%	9.5%	6.0%	4.5%	13.4%	2.0%	3.0%	5.0%	3.0%	4.0%	7.0%	5.0%	25.9%
Penn (*n* = 31)	38.7%	16.1%	16.1%	3.2%	3.2%	22.6%	9.7%	6.5%	6.5%	0.0%	6.5%	16.1%	9.7%	0.0%	9.7%	12.9%	54.8%
Shen (*n* = 142)	9.9%	3.5%	5.6%	4.9%	1.4%	5.6%	3.5%	4.9%	16.9%	1.4%	2.1%	2.8%	1.4%	4.9%	7.0%	2.1%	20.4%
Sha (*n* = 28)	17.9%	10.7%	7.1%	0.0%	3.6%	14.3%	14.3%	0.0%	3.6%	7.1%	3.6%	3.6%	3.6%	3.6%	3.6%	10.7%	21.4%
All GCB (*n* = 55)	30.9%	18.2%	7.3%	0.0%	3.6%	20.0%	12.7%	3.6%	1.8%	1.8%	0.0%	7.3%	9.1%	5.5%	5.5%	14.5%	34.5%
All non-GCB (*n* = 65)	7.7%	1.5%	7.7%	9.2%	3.1%	7.7%	1.5%	1.5%	26.2%	1.5%	6.2%	4.6%	0.0%	3.1%	10.8%	1.5%	26.2%

## Discussion

Although considered one disease entity, cases of LBCL demonstrate substantial genomic heterogeneity, which may provide insights into differential outcomes following receipt of first-line immunochemotherapy as well as subsequent lines of therapy for those patients who experience disease progression. Our analysis of *MYC* rearrangement/*MYC–BCL2* double-hit status as well as predicted function-altering genomic variants aimed to address these issues. From a cohort of nearly 700 patients captured from three series, spanning three continents with FISH and NGS performed on their initial biopsy, we identified genomic alterations that were associated with significantly higher odds of disease progression by 24 months after diagnosis, as well as present at a high frequency in cases of disease progression by 24 months independent of frequency in the entire cohort.

Of note, we chose 24 months from diagnosis as the progression time cutoff because of the fact that this was the progression-free survival primary endpoint used in recently published randomized-controlled trials for patients with newly diagnosed LBCL treated with standard therapies (6, 7) and that genomic features of an initial biopsy are more likely be concordant with those from biopsies performed at the time of first progression occurring ≤24 months compared with >24 months after initial diagnosis (8). Although it would be ideal to perform a repeat biopsy with genomic analysis at the time of disease progression, it may not always be feasible or clinically appropriate to obtain if the resulting delay in start of next treatment may result in harm to the patient; therefore, the initial biopsy may be the only source of genomic information for many cases.

Findings did not necessarily persist when series were analyzed individually, which may be not only due to smaller sample sizes than that of the entire cohort as well as patient selection but also due to potential biological differences in LBCL cases based upon race/ethnicity as patients were treated in the United States (Penn), the United Kingdom (Sha), and China (Shen). This is supported by differences in frequency of genomic alterations as well as distribution of GCB versus non-GCB COO across series. Understanding the molecular heterogeneity of LBCL is important when considering therapeutic options for subpopulations of patients with R/R LBCL.

The fact that *MYC* rearrangement and *TP53* mutations predicted for higher odds of disease progression by 24 months and that almost all disease progression events for cases harboring these features occurred by 12 months may suggest why patients with early disease progression of LBCL tend to respond poorly to salvage immunochemotherapy followed by high-dose chemotherapy with autologous stem cell transplantation (HDC/ASCT) if demonstrating chemosensitive disease as per the CORAL study (9). *MYC* rearrangement was shown to predict for poor response/survival in patients treated with this therapy in the CORAL study (10); however, it is not known whether *TP53* mutation, which is a recognized marker of resistance to cytotoxic chemotherapy, carries the same prognostic significance in this treatment setting. It is interesting to note that 60% of cases of disease progression by 12 months harbored neither *MYC* rearrangement nor *TP53* mutation. Although clinical trials have demonstrated improved survival for patients with LBCL with early disease progression randomized to receive CD19-directed chimeric antigen receptor–modified T-cell products compared with salvage immunochemotherapy followed by HDC/ASCT if demonstrating chemosensitive disease, favorable long-term survival outcomes may be achieved by patients treated with HDC/ASCT even if experiencing early disease progression (9). Therefore, investigating the impact of genomic features on outcomes following receipt of salvage immunochemotherapy may improve our ability to predict which patients with R/R LBCL may benefit from this potentially curative treatment.

*TP53* mutations were the most frequently recurring genomic alteration in all progression cases (26%). In addition to potentially predicting for resistance to cytotoxic chemotherapy in the R/R setting, *TP53* mutations may also predict for inferior response and survival following treatment with CD19-directed chimeric antigen receptor–modified T-cell products (11). Hypomethylating agents may overcome downstream effects of nonfunctional P53 protein (12), and investigation of this therapy in patients with R/R LBCL with *TP53* mutation should be considered.

The frequency of *MYC* rearrangement was high in all progression cases (15%) and specifically in those of GCB COO (31%), although this may be underrepresented in this analysis because of a low proportion of patients receiving treatment with dose-adjusted EPOCH-R, which is commonly used for the treatment of *MYC*–*BCL2* DHL. Small molecules inhibiting PI3K/histone deacetylases (13) have demonstrated moderate efficacy in the treatment of R/R *MYC*-rearranged LBCL and bromodomain and extraterminal motif proteins (14) in the treatment of MYC-overexpressed LBCL. Interestingly, *MYC* expression is reported to promote CD19 expression and vice versa in lymphoma xenografts (15), and further analysis of outcomes following treatment with CD19-directed therapies in patients with R/R *MYC*-rearranged LBCL could be explored to understand if these are of particular benefit to this population.

Mutations in *CREBBP* (20%) and *EZH2* (13%) were common in all progression cases of GCB COO, and lymphomas harboring alterations in these epigenetic modifiers may respond to histone deacetylase inhibitors (16) and enhancer of EZH2 inhibitors (17), respectively. Additionally, mutations in *MYD88* (26%), *CD79B* (9%), and *TNFAIP3* (11%) in all progression cases of non-GCB COO are noteworthy given the efficacy of the Bruton tyrosine kinase inhibitor ibrutinib in R/R activated B-cell LBCL, which seems to be more favorable in tumors with *CD79B* with or without *MYD88*^L265P^ mutations but less favorable in tumors with *TNFAIP3* mutations (18). Further evaluation of the efficacy of small-molecule inhibitors in patients with R/R LBCL based upon genomic features can be explored and potentially lead to rational combinations/sequencing of these agents with others, which may result in more immediate disease control.

Finally, mutations in *TNFRSF14* (15% in progression cases of GCB COO) and *B2M* (8% in all progression cases) may promote lymphomagenesis by affecting the immune system response to lymphoma. Mutations in *TNFRSF14* may lead to a tumor-supportive microenvironment through an increase in the production of cytokines recruiting T follicular helper cells (19), and mutations in *B2M* may lead to loss of expression of human leukocyte antigen class 1, which impairs CD8^+^ cytotoxic T-cell binding (20). These and other mutations affecting the immune system response to LBCL may result in lesser benefit from T cell–based therapies in patients harboring such features and should be further explored as predictors of response to these therapies.

Strengths of our analysis include a large sample size of patients derived from a combined multinational cohort with genomic information. Also, reporting of mutations predicted to alter protein function is important to help standardize results of mutation analysis performed with different assays and/or characterized through different variant reporting procedures, as well as identify mutations that are more likely to be of pathologic and therapeutic significance. Limitations include lack of a validation cohort, although this was not obtainable given both the infrequent performance of mutation analysis on LBCL tumors in clinical practice and infrequent reporting of progression events in many publicly available LBCL cohorts. The fact that only 15 genes were common to the panels used in the three studies included in this analysis suggests that there is an opportunity to standardize genomic testing performed on LBCL tumors. Finally, the absence of specific genomic information available in our dataset (e.g., *MYC* translocation partner and *TP53* variant allele frequency) precluded us from performing further analysis as to determine why genomic features predictive of disease progression were also present in a modest proportion of cases without disease progression.

In conclusion, genomic features of LBCL cases may predict the development of disease progression as well as inform efforts to understand their ability to serve as biomarkers for response to therapies, which could be used to treat patients with LBCL who experience disease progression and could lead to the development of more effective treatment options for subsets of patients with LBCL. Ongoing efforts to standardize genomic assays and their interpretation can increase the clinical utility of this testing for cases of LBCL.

## Supplementary Material

Supplementary Data All Supplementary Data
